# The connection between the experience of the disease and perceptions about COVID-19 in patients

**DOI:** 10.1192/j.eurpsy.2024.1062

**Published:** 2024-08-27

**Authors:** E. V. Deshchenko, J. E. Koniukhovskaia, O. B. Stepanova, I. M. Shishkova, E. I. Pervichko, O. V. Mitina, E. R. Semenova

**Affiliations:** ^1^ Lomonosov Moscow State University; ^2^Higher School of Economics, Moscow; ^3^Ryazan State Medical University, Ryazan, Russian Federation

## Abstract

**Introduction:**

Since the COVID-19 pandemic has had a serious impact on the psychological state of the population, the individual experience of COVID-19 disease may affect the content of perceptions about coronavirus in those who have been ill with it.

**Objectives:**

The aim of the research was to study the connection between patients’ experience of the disease and their perceptions about COVID-19.

**Methods:**

A Short questionnaire of Disease Perception (E. Broadbent) was used to study patients’ perceptions about COVID-19 disease. The wording “disease” was replaced with “COVID-19 disease”.

The study was conducted from January 2021 to November 2022. The sample consisted of 390 patients (64 men and 326 women), whose average age was 28.58±10.74.

**Results:**

The subjective assessment of the duration of COVID-19 disease and its impact on the patient’s life is higher if the patient is still sick with COVID-19 (r=0.340, p=0.008; r=0.312, p=0.000), in a more severe form (r=0.341, p=0.000; r=0.298, p=0.000), less satisfied with the attitude of medical workers during illness (r=0.151, p=0.003; r=0.143, p=0.005), more afraid for the health of their loved ones (r=-0.194, p=0.000; r=-0.181, p=0.000). At the same time, greater concern about COVID-19 and a greater assessment of its impact on the emotional state is associated with patients’ fear for the health of loved ones (r=-0.267, p=0.000; r=-0.242, p=0.000) and more severe course of the disease (r=0.107, p=0.035; r=0.126, p=0.013). Less sense of control in a COVID-19 disease situation is associated with a more severe course of the disease and greater fear for the health of loved ones (r=-0.174, p=0.001; r=0.154, p=0.002).

**Conclusions:**

Thus, whether the patient has recovered after COVID-19 or not yet, how severe this disease was, how satisfied he was with the attitude of medical workers towards him during the illness and how much he fears for the health of loved ones during the pandemic, is related to such perceptions about COVID-19 disease as an assessment of the disease duration, its impact on life, emotional state, concern about one’s own illness and understanding of its nature.

Disclosure: Research is supported by the Russian Science Foundation, project No. 21-18-00624.

**Disclosure of Interest:**

None Declared

